# Design and Analysis of Electrodes for Electrostimulation (TENS) Using the Technique of Film Printing and Embroidery in Textiles

**DOI:** 10.3390/s21144789

**Published:** 2021-07-13

**Authors:** Ewa Skrzetuska, Daria Michalak, Izabella Krucińska

**Affiliations:** Institute of Material Science of Textiles and Polymer Composites, Faculty of Material Technologies and Textile Design, Lodz University of Technology, 116 Żeromskiego Street, 90-924 Lodz, Poland; 209579@edu.p.lodz.pl (D.M.); izabella.krucinska@p.lodz.pl (I.K.)

**Keywords:** electrodes, carbon nanotubes, graphene, silver, screen printing, machine embroidery

## Abstract

This article describes the development of transcutaneous nerve stimulating electrodes (TENS) by means of electrically conductive ink and conductive yarn. The scope of work covered a selection of three types of knitwear with a similar surface weight with different raw material composition. Stimulating electrodes were made by means of film printing and machine embroidery. The electrodes were verified after friction tests, washing, and mechanical deformation. Each process was followed by a check of the resistive properties and assessment of the sensations in order to evaluate their performance. Tests of the surface resistance of research materials confirmed the possibility of preparing textile electrodes for electrostimulation with the use of the film-printing technique and machine embroidery. Resistance of the electrodes made on all types of substrates ranged from approximately 1.00 × 10^−2^ Ω to around 2.00 × 10^2^ Ω, while the electrodes are commercially available at the level of approximately 3.5 × 10^5^ Ω. This paper underpins the validation of the conclusion that operational processes do not adversely affect the functioning of the developed textile electrodes.

## 1. Introduction

The human epidermis is characterized by a resistance of thousands of ohms, whereby wetting significantly lowers that resistance. Ohm resistance as well as capacitive resistance affect the overall tissue impedance, which decreases along with the increase in pulse frequency. In the light of this fact, in transcutaneous electrical nerve stimulation (TENS), attention must be given to the thickness of the skin and the fact that the nerves are situated in the vicinity of the skin and adipose tissue. Smaller skin resistance is observed when short- and high-frequency pulses are used [1].

Nerve electrostimulation is a common method of physical therapy used in humans to restore motor function, enhance peripheral blood circulation, boost muscle strength, and help burn fat. It is desirable that electrostimulation therapy devices be comfortable and can be worn in everyday clothing, as this would enhance the rehabilitation of patients [2,3,4,5,6,7,8].

Transcutaneous electrical nerve stimulation (TENS) is a method through which it is possible to decrease pain sensation through the use of low-frequency currents. Pain perception can be reduced thanks to electrostimulation of the sensory fibers. Apart from its analgesic effect, TENS can also cause muscle relaxation and stimulation, as well as increase the amount of blood reaching the tissues of the body [9]. Transcutaneous electrical nerve stimulation is a commonly used nonpharmacological and noninvasive pain treatment. While a number of clinical trials have testified to the effectiveness of TENS in treating pain, there is still much controversy over what conditions should be used. By design, TENS reduces pain through peripheral and central mechanisms. Centrally, sites in the spinal cord and brainstem that use opioid, serotonin, and muscarinic receptors are activated by electrostimulation. Peripherally, opioid, and α-2 noradrenergic receptors at the site of TENS application are involved in TENS-induced analgesia [9,10].

Simplifying, TENS employs two mechanisms, i.e., gate control and endorphin release [9].

In the case of the former, the reduction of pain sensation stems from the action of a higher-frequency current, i.e., 50–100 Hz, on the mechanoreceptors in skin, the information of which is carried by the medullary Aβ nerve fibers. The impulse from the skin reaches the posterior horns of the spinal cord, and due to the large diameter of the Aβ fibers, it quickly goes to the brain. During this time, the pain information invoked by the nociceptive neurons of smaller diameter, i.e., the Aδ and coreless C fibers, is blocked upon reaching the posterior horns of the spinal cord. Because the fibers have different diameters, the magnitude of the pulse carried by them varies. When two different pieces of information occur in the posterior horns of the spinal cord, it is only the more powerful one that moves further. Therefore, the use of TENS closes the pain gate and makes further migration to the brain impossible [9,10,11,12,13,14].

On the other hand, the latter method uses currents with a frequency between 2 and 10 Hz. This time, a change of the parameters of the acting current opens the gate for the pain impulse, which allows it to reach further parts of the central nervous system. However, the action of current is not indifferent. There is a release of compounds that inhibit pain, but it occurs only at the brain stage and includes endorphins, which are endogenous opioids [9,10,11,12,13,14].

There is a division of the types of currents used for treatments, which are broken down into zero-frequency galvanic current and impulse currents with a frequency range not exceeding 1 kHz. Other types are medium- and high-frequency currents. This division only applies to physical therapy and differs from the technical one; however, it is widely recognized [9]. The types of transcutaneous electrical nerve stimulation are presented in Table 1. Any effect that a direct current may have is only possible when its intensity or direction is changed accordingly. This change must be sufficiently quick and substantial, otherwise the use of galvanic current fails to invoke any changes in relation to the excitability of nerves [9].

Proper limitation of the direct current allows for the obtaining of a pulse current. It is possible to achieve a number of different variations of the pulse current by altering parameters such as the pulse rise time (t_n_), duration (t) and fall (t_op_), amplitude, and frequency. Pulse currents can be unidirectional, unipolar, and single phase, but also bidirectional, bipolar, and two phase. Changing the three parameters of the impulse, i.e., shape, amplitude, and frequency, may lead to achieving a different effect of the stimulus [9].

Transcutaneous electrical nerve stimulator (TENS) has been used successfully in acute and chronic pain for several decades. TENS causes a light touch and pressure sensation on the skin and has been applied in reducing both chronic (such as osteoarthritis, phantom limb pain, and neuropathic pain syndromes) and acute pain (such as lacerations, fractures, hematomas, contusions, and postoperative pain) [15].

There are several types of electrodes used in electrotherapy, i.e., metal, silicone, self-adhesive, special purpose, and textile ones [16,17,18].

Metal electrodes, which take the form of thin plates made from tin or aluminum, are used together with viscose pouches and elastic straps to ensure the greatest possible safety for patients. The use of these metals is obvious due to their good conductivity [16,17].

Silicone electrodes are made from conductive silicone or silicone-protected carbon rubber. They have a significant durability even with very frequent use and can be disinfected. Their proper positioning requires additional elements in the form of a viscose bag and an elastic belt, as they have no layer that would stick to the skin [17,18].

Self-adhesive electrodes have special gels that ensure good adherence to the skin during electrostimulation treatments, as well as gels that enable proper joining of subsequent layers of the electrode. The structures of some are fit with a special high-density carbon mesh or silver. Self-adhesive electrodes are not as durable as silicone ones and lose their function after about 30 uses. High-quality electrodes can be used a greater number of times, because their gel regains its adhesive properties after contact with water [16,17,18].

Textile electrodes are characterized by better efficiency compared to self-adhesive ones. Their structure is enriched with special conductive fibers, which usually contain silver. Textile electrodes are suitable for patients who are allergic to gels, with irritated skin that sweats intensely or is covered with thick hair. Thanks to their perfect adhesion to the body, they do not cause unpleasant stinging or burning sensations during therapy. They are used for electrostimulation of the knees, elbows, feet, and hands. The market also offers system electrodes designed to stimulate various parts of the body, such as the spine, thighs, or arms, which are made in such a way that the electrode placement is ideally suited to a specific body area [16,17,18].

An example of clothing with built-in thermotherapy and a TENS device was developed by Professor Lee et al. who created it to alleviate painful menstruation. The research comprised an attempt to use smart clothing as a method for alleviating painful menstruation, which is an obstacle to many social activities for women. The TENS device is designed as a belly band, which makes it imperceptible to others when worn under outer garments; the size has been minimized and attached to the underwear for easy use anytime and anywhere. The current problem is the limitation of minimization, but as battery technology continues to develop, we can expect much smaller devices [19].

Transcutaneous superficial electrical stimulation (TES) is a widely used technique for treating muscle atrophy, muscle strength training, endurance training, pain management, functional movement therapy, and restoration of motor function. The Keller team unveiled a new TES technology based on multichannel stimulation that allows real-time spatial and temporal changes in electric current density on the skin surface and deeper layers of tissue. The described approach can generate better muscle selectivity and enhanced muscle activation patterns compared to state-of-the-art TES systems that operate with predetermined electrode positions. The publication demonstrated that it was possible to generate selective straightening movements of the fingers and wrists in poststroke patients that were strong enough to overcome flexion hyperactivity [20].

Another article discussed the design and implementation of a wireless wearable therapy device for pressure ulcer prevention. As a replacement for conventional patient repositioning methods and air mattresses, the system provides electrical stimulation (ES) at pressure points in the gluteal muscles. The whole system includes a built-in electrical stimulation device with flexible electrodes that stick to the skin. Users can adjust electrotherapy parameters via a smartphone application connected wirelessly to the clothes. The presented wearing set, optimized for muscle contraction and pressure ulcer prevention, has the potential to assist bedridden patients [21].

Research by Goncu and Berk describes the design process of a pain management system using embroidered electrodes, which required not only technical optimization but also understanding the issues of users related to the use of conventional TENS devices. The proposed end product is a user-friendly, textile-based electronic pain management system suitable for major pain areas such as the knee, elbow, and neck [22].

Kim and Cho developed an e-fabric-based smart glove with embedded textile electrodes. One side of the electrodes is a conductive clip that is used to connect to one end of a transmission line [23].

The authors decided to focus on the production of electrodes for transcutaneous electrical nerve stimulation on textile substrates, which are used to constitute a personalized product in the form of a textronic garment using techniques commonly considered decorative. Textronics is a dynamically developing segment of textile products which combines elements of electronics and textiles. Manufacturing textronic systems is not an easy task. Garments must meet certain properties; therefore, attention must be paid to a number of factors. When designing clothing, one must not forget about the requirements accompanying electronic products, including accuracy, measuring range, preservation of selected textile products, such as low weight, flexibility, and the applicable rules of material engineering. The correct selection of textiles and electronic systems, as well as their mutual integration, is a great challenge for scientists. The increase in technology development goes hand in hand with an improved quality of the systems produced. Textronic products are mainly produced using everyday clothes combined with a miniaturized electronic system, sensors, and power system.

All this is made possible with sensors. Characteristic features of some raw materials used to make textiles include piezoelectric properties, electrostatic qualities, and shape memory. Materials that employ these functions are called smart and combine the functionalities of both a sensor and an actuator.

The authors selected three different textile substrates of similar surface weight, as well as two printing pastes and one electrically conductive yarn. Next, they performed functional tests such as washing, friction, and stretching processes, and verified their performance in terms of conductive properties.

## 2. Materials and Methods

The aim of the research was to measure the electrical resistance of the developed textile electrode for transcutaneous electrical nerve stimulation before and after the utility processes. The thesis posed was that film printing and machine embroidery would allow one to obtain textile electrodes for electrostimulation, which are durable in usable processes. It was assumed that the electrodes must be flexible, resistant to deformation, and not cause discomfort. For this purpose, rectangular electrodes were designed on the surface of which electrically conductive layers were placed, both with full and spiral filling. The aim of the project was to determine the best method of electrode manufacturing by means of textile techniques, which are durable in the processes of use.

In the paper, we used two printing pastes with conductive properties. The former was an aqueous dispersion of carbon nanotubes from Nanocyl under the trade name AquaCyl AQ0301, which included NC7000TM series multiwalled carbon nanotubes (MWCNT) with a diameter of 9.5 nm and a length of 1.5 μm in the amount of 3% by weight. Surfactants were used as an additive to improve dispersion and stability. The second one was a water dispersion of Nanocyl carbon nanotubes under the trade name AquaCyl AQ0301, enriched with graphene granules (GO) purchased from Graphene Supermarket. Graphene MO-1 was in the form of 5–30 nm multilayer granules. The composition contained 3% by weight of carbon nanotubes and 2.5% by weight of graphene.

Furthermore, for embroidery, we used Noble metallized yarn under the trade name X-STATIC^®^, made of 85% polyamide silk, which was covered with a 15% pure silver coating.

**Figure 1:**
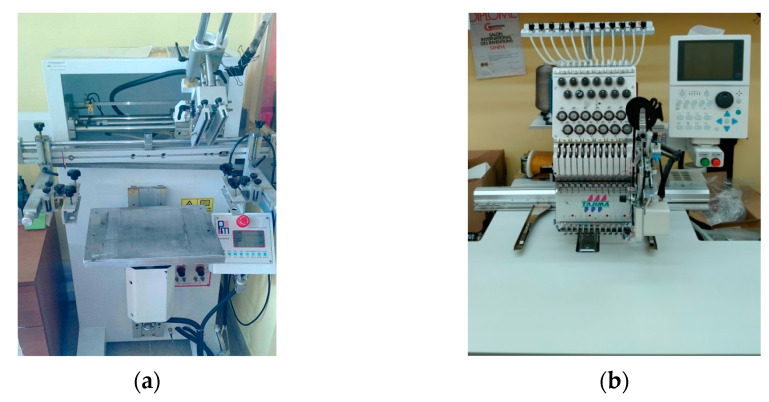
(**a**) The MS-300 machine used in the screen-printing method, (**b**) “Tajima” embroidery machine, model: “TMEX-C1201”.

In the film-printing technique, we used SAATI’s 60T polymer mesh with an aluminum frame on the MS-300 machine (Figure 1a). Embroidery was made by means of the Tajima TEMX-1201 embroidery machine (Figure 1b), which was loaded with the completed design of the embroidery pattern. Stability of the embroidery process was ensured by using interlining as a base for the knitwear.

Three knitwear differing in terms of the raw material composition (natural, artificial, and synthetic fibers) with a similar mass per unit area were used as textile substrates for the production of TENS prototypes:

- Composition: 97% cotton, 3% elastane. Surface weight: 217 g/m^2^.
- Composition: polyester 95%, elastane 5%. Surface weight: 240 g/m^2^.
- Composition: viscose 96%, elastane 4%. Surface weight: 262 g/m^2^.

**Figure 2:**
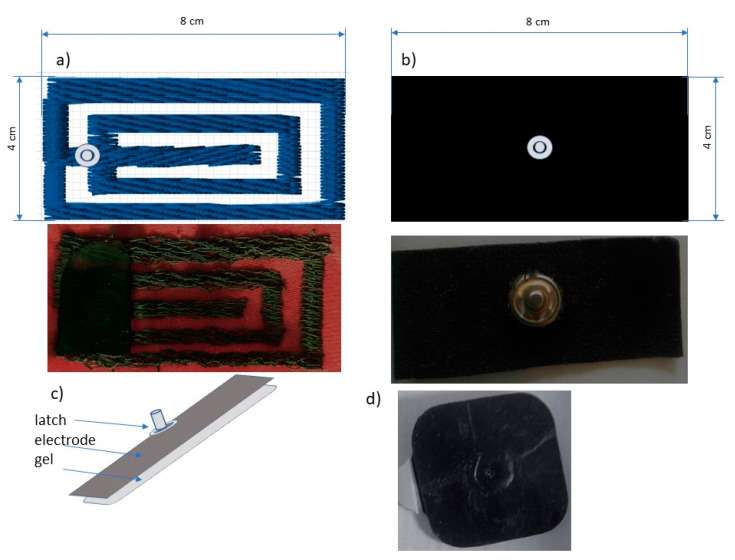
Pattern of electrode made by (**a**) machine embroidery technique, (**b**) film printing, (**c**) electrode cross-section, and (**d**) commercial electrode.

Prints and embroideries were made in the format 4 cm × 8 cm (Figure 2). In the middle, there is a latch compatible with the TENS simulation device. The inside of the electrodes may have a gel applied or the skin may be smeared with a gel to prevent skin irritation.

Commercial electrodes with a silver-plated layer on a nonwoven gel-layer were used as reference electrodes.

The core aim of the research was to conduct surface resistance tests. Surface resistance was tested in accordance with PN-EN 1149-1 Protective clothing—Electrostatic properties, using a Keithley 2000 digital multimeter, which was used to verify changes in the resistance of research materials after utility processes.

First, the surface mass was tested in accordance with the PN-EN 12127:2000 standard for Textiles—flat textile products—determination of mass per unit area using small samples and thickness tests. Thicknesses were measured according to PN-EN ISO 5084 Textiles—determination of the thickness of textile products. For the purpose of testing, we used the Check Line thickness gauge, model J-40-V. Five measurements were taken for each type of knitted fabric and each test material. The area of the bead used was 2000 mm^2^ and exerted a pressure of 1 kPa. Next, we performed air permeability tests in accordance with PN-EN ISO 9237:1995 Textiles—determination of permeability of fabrics to air. For this purpose, we used the measuring device FX 3300. The air flowed perpendicularly through the tested knitwear with an area of about 20 cm^2^, with a pressure drop of 100 Pa.

Subsequent research was undertaken to simulate the functional processes of clothing products, such as friction, stretching, and washing.

Resistance to friction was tested in accordance with PN-EN ISO 12945-2 Textiles—determining the tendency of the surface of a flat product to pilling and pilling—Part 2: Modified Martindale method, by means of the M235 Martindale device. Three circles with a diameter of 140 mm were cut from each of the three types of textiles and placed in a holder as a grinding wheel during the test. Next, one circle, also 140 mm in diameter, was cut from each of the nine types of material modified by film printing and embroidery and fixed on the machine table. The test was carried out under a load of 595 g. In the next step, instead of assessing the pilling degree, the change in surface resistance of all nine samples was assessed after 300 strokes and after 900 strokes.

The study of surface resistance changes due to stretching was carried out according to our own method. For this purpose, we used the Instron 5944 testing machine. One 300 mm × 50 mm strip was cut from each type of modified knitted fabric. Instron Bluehill developed a method in which the distance between the clamps was set to 200 mm and the tensile distance to 20 mm to stretch the sample by 10% of its length. The number of cycles was set to 100. Tensile speed was 200 mm/min. Surface resistance tests were performed successively after 100, 300, and 500 stretching cycles.

Washing resistance was tested according to the PN-ISO 105-C06:1996 standard: Textiles—color fastness tests—Color fastness to domestic and commercial laundering, using the Ugolini laboratory dye. Deviation from the norm was recognized as the lack of evaluation of the samples according to the gray scale after washing, while their surface resistance was measured. From each of the nine types of test materials, two 100 mm × 50 mm samples were prepared, and the surface resistance was measured for each one. Samples were washed in conditions specified in the standard as A1S with the use of steel balls. The washing agent used was ECE PHOSPHATE REFERENCE DETERGENT B. All samples were washed separately without acidification. After the test specimens had dried, the changes in their surface resistance after one wash and five washes were assessed.

The last stage of the research work was to test ready-made electrodes for transcutaneous electrical nerve stimulation. The electrodes were made according to our own method. Samples for creating the electrodes were prepared from nine starting materials, nine samples after 900 pilling strokes, nine samples after 500 stretching cycles, and nine samples after 5 washes, by cutting 40 mm × 80 mm strips. Therefore, 36 different test combinations were available. Next, all the obtained samples were padded with nickel nappers with the aid of a napping machine, so that the head of the nap was on the unprinted side of the material in the case of samples made by film printing. Thereafter, commercially available gel pads were glued to each sample. The electrodes made in this way were glued to a metal base, and their surface resistance was measured with a Keithley 2000 digital multimeter by connecting one wire to the snap head and the other to the metal plate.

Moreover, using a commercially used electrostimulation device, the correct operation of the developed electrodes was verified in comparison with the commercially available ones.

## 3. Results and Discussion

As a result of the tests carried out under normal climate conditions, after a 24-h acclimatization of the samples, the following values were determined:

- surface mass,
- thickness,
- air permeability,
- surface resistance after utility processes,
- surface resistance of the electrodes.

It follows from the analysis of data in Table 2 that the surface weight for each type of textile material increased after the alterations. Considering all nine combinations, the highest increase in surface weight by 173 g/m^2^ was observed for embroidered viscose, while the smallest by 10 g/m^2^ was noted for viscose printed with carbon nanotubes and graphene. The highest increase in area weight in the case of film printing was observed for printing with the use of carbon nanotubes on viscose by 52 g/m^2^. The embroidery process contributed to the greatest increase in the surface weights of textile materials. Embroidery increased the area weight of cotton by 158 g/m^2^ and that of polyester by 125 g/m^2^.

It follows from the analysis of Table 3 that the greatest increase in thickness, with a value equal to 2.43 mm, was recorded for the embroidered viscose. The largest decrease in thickness by 0.13 mm was observed for viscose printed with carbon nanotubes and graphene. Film printing with the use of carbon nanotubes decreased the thickness of cotton by 0.03 mm and that of polyester by 0.05 mm, while the thickness of the viscose sample increased by 0.26 mm. Additionally, analysis of the coefficient of variation implies that the most uniform layer was made on cotton (V = 1.2%), then on polyester (V = 2.6%), followed by viscose (V = 4.1%). Printing with a paste with carbon nanotubes and graphene contributed to a decrease in the thickness of cotton and viscose by 0.02 and 0.13 mm, respectively, while no difference was noted for polyester. As for the uniformity of printing with this type of paste, the best results were obtained for polyester (V = 1.2%), and then for cotton (V = 3.3%), while the most uneven layer was made on viscose (V = 4.2%). Embroidery increased the thickness of cotton by 1.56 mm and that of polyester by 1.75 mm. Reduction of thickness in the case of film printing may be dictated by the fact that the sample was crushed during printing, which may indicate a permanent deformation resulting from the pressure of the screen and squeegees on the printed material.

By interpreting the data in Table 4, it can be seen that the greatest increase in air permeability was equal to 29 mm/s and occurred for the cotton knitwear after embroidery. On the other hand, the highest decrease in air permeability was observed for polyester knitwear printed with carbon nanotubes; it was 292 mm/s. Printing with a paste with carbon nanotubes resulted in a reduction of air flow through cotton and viscose knitwear by 127 and 92 mm/s, respectively. Similar drops in air permeability appeared after using the paste with carbon nanotubes and graphene. Here, too, the highest decrease was recorded for polyester knit, then for cotton knit and viscose, by 285, 169, and 65 mm/s, respectively. Interpretation of the coefficient of variation shows that the most even layer obtained after the film-printing process was achieved with polyester covered with a paste with carbon nanotubes and graphene. In the case of embroidery, there were merely slight increases in air permeability of 20 mm/s for viscose and 16 mm/s for polyester. The postprinting decrease in air permeability is due to the fact that the free spaces between the fibers have been filled with printing paste. On the other hand, the increase in air permeability after embroidery stems from the fact that the needle pierced the knitted structure, thereby creating additional air spaces.

It follows from the data in Table 5 that the surface resistance of all samples increased compared to the initial values. The largest recorded resistance difference equal to 6.02 × 10^4^ Ω was exhibited by a polyester knitwear printed with carbon nanotubes and graphene, thus achieving the highest resistance among all samples reaching 7.23 × 10^4^ Ω. In the case of cotton knitwear printed with carbon nanotubes, the resistance first dropped to 1.11 × 10^4^ Ω after 300 strokes, but after another 600 strokes, the resistance reached the value of 3.57 × 10^4^ Ω, which is higher than the initial figure. Conversely, the viscose knitwear was printed with carbon nanotubes, and the cotton fabric was printed with carbon nanotubes and graphene. In that case, the resistance increased after 300 strokes; however, when it reached 900 strokes, it decreased but did not fall below the initial value. The material printed with the paste with carbon nanotubes that had the highest difference in resistance was a polyester knitwear with a result of 1.29 × 104 Ω. The smallest difference in resistance equal to 0.5 × 10^3^ Ω was observed for viscose knitwear covered with carbon nanotubes. Considering the postfriction resistance for materials after film printing, it can be seen that the differences were greater in the case of materials printed with a paste with carbon nanotubes and graphene. The embroidered samples exhibited practically no change in resistance compared to what happened with the printed test materials. The biggest difference of 6.1 Ω was demonstrated by a polyester knitwear sample. It follows that friction deteriorates the properties; however, it may happen that these properties are improved. It may result from the fact that during the process, some of the printing paste present on better covered areas is rubbed into the voids, thereby increasing the conductivity of samples. The samples of commercial electrodes were damaged during the operational tests.

Data from Table 6 implies that the surface resistance of all test materials increased successively after 100 and 300 stretching cycles, reaching the highest values after 500 stretching cycles. The biggest difference between the initial resistance and the resistance after 500 cycles is 4.51 × 10^4^ Ω, which is observed for cotton knit fabric printed with carbon nanotubes. In turn, the smallest difference in resistance for printed samples equal to 1.71 × 10^4^ Ω was exhibited by a viscose knit covered with carbon nanotubes. Knitwear with embroidery showed practically no shift in resistance after stretching, while the greatest difference of 2.33 Ω was recorded for cotton knitwear. It was observed that cotton knitwear exhibited the greatest increase in resistance compared to the initial resistance for each of the three types of conductors. Stretching deteriorates conductive properties, which is the result of breaking single conductivity paths during the action of mechanical forces. The samples of commercial electrodes were damaged during the operational tests.

It follows from Table 7 that the surface resistance increased significantly after five washes compared to that after one wash for each of the printed samples. Polyester knitwear printed with carbon nanotubes is characterized by the greatest difference in resistance, achieving the highest results of all at 3.21 × 10^6^ Ω. The smallest difference after five washes, with the value of 6.93 × 10^4^ Ω for the printed samples, was observed for viscose knit fabric with carbon nanotubes. Considering only the printed samples, it appears that both those printed with a paste with carbon nanotubes and those coated with a paste with carbon nanotubes and graphene exhibited the highest increases in resistance first for polyester knit, then for cotton and finally for viscose. Washing did not cause any considerable differences with the samples with embroidery. There was practically no change in these results, whereby the largest change of 7.9 Ω was recorded for cotton knitwear. The samples of commercial electrodes were damaged during the operational tests.

Differences in the values of surface resistance in electrodes and test samples are caused by the use of commercial gel pads and a different method of measuring resistance.

Table 8 demonstrated that the electrodes made have a lower surface resistance value than commercial electrodes. Furthermore, even the utility processes to which the electrodes have been subjected exhibited less resistance. The samples of commercial electrodes were damaged during the operational tests.

**Figure 3:**
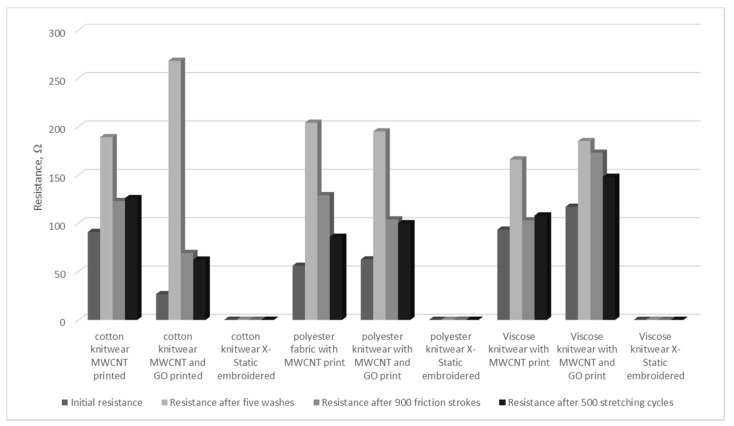
Dependence of changes in the surface resistance in the produced electrodes.

It follows from Table 8 and Figure 3 that the highest surface resistance of 2.04 × 10^2^ Ω was achieved by an electrode made from polyester knitwear printed with a paste based on carbon nanotubes, which was washed five times. In the case of prints with a paste based on carbon nanotubes, electrodes on cotton and viscose knitwear, washing increased resistance to 1.89 × 10^2^ Ω and 1.66 × 10^2^ Ω. For these samples, friction caused the greatest increase in resistance in an electrode made on polyester knitwear by 7.3 × 10^1^ Ω. In case of the cotton knitwear electrode, friction increased the resistance by merely 3.2 × 10^1^ Ω and in case of the viscose electrode by 9.6 Ω. Stretching enhanced the electrode resistance on cotton knitwear to 1.26 × 10^2^ Ω. Electrodes on viscose and polyester knitwear displayed an increase in resistance by 3.0 × 10^1^ Ω and 1.46 × 10^1^ Ω.

For prints made on the basis of carbon nanotubes and graphene, the highest value of resistance after washing, equal to 3.20 × 10^2^ Ω, is observed for an electrode on polyester knitwear. In the case of the electrode on cotton and viscose knitwear, the resistance increased by 1.33 × 10^2^ Ω and 6.8 × 10^1^ Ω. Following the pilling process, the resistance of all electrodes made on the basis of carbon nanotubes and graphene increased, but the greatest increase of 2.49 × 10^2^ Ω was noted for polyester knitwear, followed by viscose 1.73 × 10^2^ Ω and cotton 1.04 × 10^2^ Ω. Stretching increased the resistance of electrodes on polyester and viscose knitwear to 1.87 × 10^2^ Ω and 1.48 × 10^2^ Ω. The electrode on the cotton knitwear increased the resistance by 3.84 × 10^1^ Ω after stretching.

The analysis of data regarding the electrodes made with embroidery reveals slight differences in the resistance values of the electrodes for all types of textile materials and after all the utility processes they have been subjected to. In the case of cotton knitwear, it was only friction that caused a drop in resistance to 1.36 × 10^−1^ Ω, while washing and stretching increased it to 1.97 × 10^−1^ Ω and 2.02 × 10^−1^ Ω. The resistance of the electrode on the polyester knitwear increased slightly after washing, friction, and stretching, i.e., by 1.00 × 10^−2^ Ω, 1.62 × 10^−1^ Ω, 1.12 × 10^−1^ Ω. In the case of the viscose knitwear electrode, the resistance decreased to 5.60 × 10^−2^ Ω only after friction, while washing increased resistance by 3.4 × 10^−2^ Ω, and stretching by 6.0 × 10^−2^ Ω.

## 4. Conclusions

Film printing and machine embroidery represent excellent technological solutions for producing electrodes for transcutaneous electrical nerve stimulation. Analysis of the research presented herein demonstrates that both of the described methods for producing textile electrodes enable a quick and noninvasive production of intelligent textiles which have proven useful in rehabilitation and exercise. That said, it should be remembered that the application of printing pastes to textile surfaces reduces air permeability, which is best illustrated by the polyester knitwear whose preprinting permeability was 580 mm/s, which later decreased to 288 mm/s in the case of paste with carbon nanotubes and 295 mm/s for prints based on carbon nanotubes and graphene. Polyester knitwear is characterized by the highest level of air permeability, i.e., breathability, among the materials tested. In addition, polyester products carry moisture away from the skin, which prevents the feeling of wetness and cold around the body. Prints made on textile materials increase surface weight and alter thickness, while embroidery increases their surface weight, thickness, and air permeability. Therefore, it should be remembered that prints and embroidery should be made in such a way as to have the least possible impact on the user’s biophysical comfort.

Utility processes such as friction, stretching, and washing enhance the surface resistance of test materials, i.e., they adversely affect their conductive properties. The application process which caused the greatest increase in surface resistance turned out to be washing. Polyester knitwear, printed with carbon nanotubes, exhibits the greatest difference in resistance for the samples after five washes, reaching the highest value of all the results at 3.21 × 10^6^ Ω. The smallest difference after five washes, with the value of 6.93 × 10^4^ Ω for the printed samples, was observed for viscose knitwear with carbon nanotubes.

Research materials made by film printing turned out to be less resistant to utility processes than those created by machine embroidery in terms of conductive properties. Embroidery made with the use of X-STATIC yarn is more resistant to friction, stretching, and washing than prints made with the use of two types of conductive pastes.

The analysis implies that viscose knitwear is the best textile material for introducing modifications by film printing. The prints made on it were the most resistant to operational processes. After five hundred stretching cycles, the resistance for viscose knitwear printed with a paste based on carbon nanotubes and graphene changed from 1.97 × 10^4^ to 4.79 × 10^4^ Ω, and after 900 friction cycles, it was altered from 1.97 × 10^4^ to 4.47 × 10^4^ Ω.

In the case of machine embroidery, the type of textile material did not significantly affect the value of surface resistance.

Comparative tests demonstrated that each of the nine produced electrodes has better conductive properties compared to commercial electrodes. Even the electrodes subjected to utility processes showed no greater surface resistance than the commercial electrode.

The gel pad used to make the electrodes allowed for their better contact with the skin and significantly lowered their surface resistance value.

That said, it must be kept in mind that intelligent products used for rehabilitation and exercises should be personalized in such a way as to ensure precise adherence to the body and not cause excessive pressure on the stimulated body areas.

In their subsequent works, the authors assume that the optimization processes of electrodes obtained by embroidery techniques are carried out, which in the design process showed the highest resistance to use. Further work is related to the refinement of embroidery patterns, such as the size, shape, and location of human symbols, as well as the use of gels on the developed electrodes.

## Figures and Tables

**Table 1:** Types of TENS.

	Conventional	Acupuncture	Burst	Intensive
Frequency [Hz]	50–200	0.5–10	80–100	60–100
Pulse time [μs]	50–250	150–250	100–300	150–250
Time of procedure [min]	30–60	20–30	20–45	15–20

**Table 2:** Values of surface masses in textile materials before and after alterations.

Measured Variant	Surface Mass, g/m^2^	Standard Deviation, g/m^2^	Coefficient of Variation, %
cotton knitwear	217.00	0.41	0.19
cotton knitwear MWCNT printed	247.00	0.82	0.33
cotton knitwear MWCNT and GO printed	258.00	0.81	0.32
cotton knitwear X-Static embroidered	375.33	1.25	0.33
polyester knitwear	240.33	1.24	0.52
polyester fabric with MWCNT print	266.67	1.25	0.46
polyester knitwear with MWCNT and GO print	256.00	1.63	0.63
polyester knitwear X-Static embroidered	365.01	0.81	0.22
viscose knitwear	262.03	0.82	0.31
viscose knitwear with MWCNT print	313.67	2.05	0.62
viscose knitwear with MWCNT and GO print	272.00	1.63	0.60
viscose knitwear X-Static embroidered	435.01	0.82	0.18

**Table 3:** Values of the average thickness of textiles before and after alterations.

Measure Variant	Thickness, mm	Standard Deviation, mm	Coefficient of Variation, %
cotton knitwear	0.86	0.01	0.90
cotton knitwear MWCNT printed	0.83	0.01	1.20
cotton knitwear MWCNT and GO printed	0.84	0.03	3.30
cotton knitwear X-Static embroidered	2.42	0.09	5.80
polyester knitwear	0.94	0.02	2.00
polyester fabric with MWCNT print	0.89	0.02	2.60
polyester knitwear with MWCNT and GO print	0.94	0.01	1.20
polyester knitwear X-Static embroidered	2.69	0.06	2.20
viscose knitwear	0.88	0.01	0.90
viscose knitwear with MWCNT print	1.14	0.05	4.1
viscose knitwear with MWCNT and GO print	0.75	0.03	4.20
viscose knitwear X-Static embroidered	3.31	0.02	0.60

**Table 4:** Results of average values of air permeability in textile materials before and after alterations.

Measure Variant	Air Permeability, mm/s	Standard Deviation, mm/s	Coefficient of Variation, %
cotton knitwear	278.00	9.00	3.10
cotton knitwear MWCNT printed	151.00	53.00	35.10
cotton knitwear MWCNT and GO printed	109.00	16.00	14.60
cotton knitwear X-Static embroidered	307.00	16.00	5.20
polyester knitwear	580.00	22.00	3.90
polyester fabric with MWCNT print	288.00	74.00	25.60
polyester knitwear with MWCNT and GO print	295.00	21.00	7.20
polyester knitwear X-Static embroidered	596.00	17.00	2.80
viscose knitwear	145.00	5.00	3.50
viscose knitwear with MWCNT print	53.00	10.00	18.90
viscose knitwear with MWCNT and GO print	80.00	15.00	19.10
viscose knitwear X-Static embroidered	165.00	14.00	8.40

**Table 5:** Results of average values of surface resistances in test materials after friction.

Measure Variant	Statistical Measure	Initial Resistance	Resistance after 300 Strokes	Resistance after 900 Strokes
commercial electrode	$\bar{x}$, Ω	3.51 × 10^5^	-	-
	σ, Ω	6.17 × 10^5^	-	-
	V, %	175.9	-	-
cotton knitwear MWCNT printed	$\bar{x}$, Ω	2.5 × 10^4^	1.11 × 10^4^	3.57 × 10^4^
	σ, Ω	9.47 × 10^3^	7.59 × 10^3^	2.54 × 10^4^
	V, %	32.1	68.2	71.3
cotton knitwear MWCNT and GO printed	$\bar{x}$, Ω	2.31 × 10^4^	5.19 × 10^4^	4.82 × 10^4^
	σ, Ω	4.94 × 10^3^	1.76 × 10^4^	2.60 × 10^4^
	V, %	21.3	34.0	54.0
cotton knitwear X-Static embroidered	$\bar{x}$, Ω	2.50 × 10^0^	5.50 × 10^0^	5.67 × 10^0^
	σ, Ω	1.12 × 10^0^	2.50 × 10^0^	1.70 × 10^0^
	V, %	44.7	45.5	30.0
polyester fabric with MWCNT print	$\bar{x}$, Ω	2.47 × 10^4^	3.03 × 10^4^	3.76 × 10^4^
	σ, Ω	6.75 × 10^3^	1.76 × 10^4^	3.23 × 10^4^
	V, %	27.4	58.1	86.0
polyester knitwear with MWCNT and GO print	$\bar{x}$, Ω	1.21 × 10^4^	6.44 × 10^4^	7.23 × 10^4^
	σ, Ω	3.18 × 10^3^	1.72 × 10^4^	2.54 × 10^4^
	V, %	26.2	26.7	35.1
polyester knitwear X-Static embroidered	$\bar{x}$, Ω	4.40 × 10^0^	8.67 × 10^0^	1.05 × 10^1^
	σ, Ω	2.06 × 10^0^	4.89 × 10^0^	2.50 × 10^0^
	V, %	46.8	56.4	23.8
viscose knitwear with MWCNT print	$\bar{x}$, Ω	3.50 × 10^3^	4.33 × 10^3^	4.00 × 10^3^
	σ, Ω	1.50 × 10^3^	1.94 × 10^3^	2.00 × 10^3^
	V, %	42.9	44.9	50.0
viscose knitwear with MWCNT and GO print	$\bar{x}$, Ω	1.97 × 10^4^	2.68 × 10^4^	4.47 × 10^4^
	σ, Ω	1.35 × 10^4^	1.28 × 10^4^	1.75 × 10^4^
	V, %	68.5	47.9	39.2
viscose knitwear X-Static embroidered	$\bar{x}$, Ω	6.00 × 10^0^	7.83 × 10^0^	8.83 × 10^0^
	σ, Ω	2.74 × 10^0^	3.53 × 10^0^	4.45 × 10^0^
	V, %	45.6	45.1	50.4

* $\bar{x}$—average, σ—standard deviation, V—coefficient of variation.

**Table 6:** Results of average values of surface resistances in test materials after the tensile process.

Measure Variant	Statistical Measure	Initial Resistance	Resistance after 100 Cycles	Resistance after 300 Cycles	Resistance after 500 Cycles
commercial electrode	$\bar{x}$, Ω	3.51 × 10^5^	-	-	-
	σ, Ω	6.17 × 10^5^	-	-	-
	V, %	175.9	-	-	-
cotton knitwear MWCNT printed		2.95 × 10^4^	4.96 × 10^4^	5.04 × 10^4^	7.46 × 10^4^
	σ, Ω	9.47 × 10^3^	2.55 × 10^4^	1.07 × 10^4^	1.79 × 10^4^
	V, %	32.1	51.4	21.3	24.0
cotton knitwear MWCNT and GO printed	$\bar{x}$, Ω	2.31 × 10^4^	4.36 × 10^4^	4.82 × 10^4^	5.29 × 10^4^
	σ, Ω	4.94 × 10^3^	1.18 × 10^4^	1.90 × 10^4^	9.05 × 10^3^
	V, %	21.3	27.1	39.3	17.1
cotton knitwear X-Static embroidered	$\bar{x}$, Ω	2.50 × 10^0^	2.83 × 10^0^	4.83 × 10^0^	4.83 × 10^0^
	σ, Ω	1.12 × 10^0^	1.34 × 10^0^	2.11 × 10^0^	2.79 × 10^0^
	V, %	44.7	47.4	43.8	57.8
polyester fabric with MWCNT print	$\bar{x}$, Ω	2.47 × 10^4^	4.10 × 10^4^	4.21 × 10^4^	4.74 × 10^4^
	σ, Ω	6.75 × 10^3^	8.87 × 10^3^	1.07 × 10^4^	8.93 × 10^3^
	V, %	27.4	21.6	25.4	18.8
polyester knitwear with MWCNT and GO print	$\bar{x}$, Ω	1.21 × 10^4^	3.36 × 10^4^	3.56 × 10^4^	3.59 × 10^4^
	σ, Ω	3.18 × 10^3^	1.28 × 10^4^	4.97 × 10^3^	9.85 × 10^3^
	V, %	26.2	38.0	14.0	27.5
polyester knitwear X-Static embroidered	$\bar{x}$, Ω	4.40 × 10^0^	4.50 × 10^0^	5.50 × 10^0^	6.17 × 10^0^
	σ, Ω	2.06 × 10^0^	2.63 × 10^0^	2.14 × 10^0^	2.79 × 10^0^
	V, %	46.8	58.4	38.9	45.3
viscose knitwear with MWCNT print	$\bar{x}$, Ω	3.50 × 10^3^	1.59 × 10^4^	1.88 × 10^4^	2.06 × 10^4^
	σ, Ω	1.50 × 10^3^	8.88 × 10^3^	1.03 × 10^4^	6.02 × 10^3^
	V, %	42.9	55.8	55.0	29.3
viscose knitwear with MWCNT and GO print	$\bar{x}$, Ω	1.97 × 10^4^	2.91 × 10^4^	3.44 × 10^4^	4.79 × 10^4^
	σ, Ω	1.35 × 10^4^	1.70 × 10^4^	8.64 × 10^3^	1.15 × 10^4^
	V, %	68.5	58.5	25.1	23.9
viscose knitwear X-Static embroidered	$\bar{x}$, Ω	6.00 × 10^0^	7.17 × 10^0^	7.67 × 10^0^	7.83 × 10^0^
	σ, Ω	2.74 × 10^0^	3.34 × 10^0^	3.30 × 10^0^	3.29 × 10^0^
	V, %	45.6	46.6	43.0	42.0

* $\bar{x}$—average, σ—standard deviation, V—coefficient of variation.

**Table 7:** Results of average values of surface resistances in test materials after washing.

Measure Variant	Statistical Measure	Resistance before Washing	Resistance after One Wash	Resistance after Five Washes
commercial electrode	$\bar{x}$, Ω	3.51 × 10^5^	-	-
	σ, Ω	6.17 × 10^5^	-	-
	V, %	175.9	-	-
cotton knitwear MWCNT printed	$\bar{x}$, Ω	2.85 × 10^4^	1.48 × 10^5^	1.60 × 10^6^
	σ, Ω	1.71 × 10^4^	3.52 × 10^4^	1.06 × 10^6^
	V, %	60.1	23.8	65.9
cotton knitwear MWCNT and GO printed	$\bar{x}$, Ω	2.47 × 10^4^	7.66 × 10^4^	2.06 × 10^6^
	σ, Ω	1.88 × 10^4^	2.40 × 10^4^	8.10 × 10^5^
	V, %	76.1	31.4	39.4
cotton knitwear X-Static embroidered	$\bar{x}$, Ω	2.50 × 10^0^	5.20 × 10^0^	1.04· × 10^1^
	σ, Ω	1.12 × 10^0^	1.33 × 10^0^	2.06 × 10^0^
	V, %	44.7	25.5	19.8
polyester fabric with MWCNT print	$\bar{x}$, Ω	2.38 × 10^4^	8.40 × 10^4^	3.21 × 10^6^
	σ, Ω	1.26 × 10^4^	3.11 × 10^4^	8.04 × 10^5^
	V, %	53.2	37.0	25.1
polyester knitwear with MWCNT and GO print	$\bar{x}$, Ω	1.81 × 10^4^	1.23 × 10^5^	2.35 × 10^6^
	σ, Ω	5.83 × 10^3^	4.72 × 10^4^	1.25 × 10^6^
	V, %	32.3	38.4	53.1
polyester knitwear X-Static embroidered	$\bar{x}$, Ω	4.40 × 10^0^	4.80 × 10^0^	7.40 × 10^0^
	σ, Ω	2.06 × 10^0^	1.33 × 10^0^	2.87 × 10^0^
	V, %	46.8	27.6	38.8
viscose knitwear with MWCNT print	$\bar{x}$, Ω	4.38 × 10^3^	2.75 × 10^4^	7.37 × 10^4^
	σ, Ω	3.43 × 10^3^	2.41 × 10^4^	4.26 × 10^4^
	V, %	78.3	87.6	57.7
viscose knitwear with MWCNT and GO print	$\bar{x}$, Ω	1.65 × 10^4^	5.88 × 10^4^	2.24 × 10^5^
	σ, Ω	1.34 × 10^4^	3.44 × 10^4^	1.57 × 10^5^
	V, %	81.5	58.6	69.8
viscose knitwear X-Static embroidered	$\bar{x}$, Ω	6.00 × 10^0^	6.80 × 10^0^	1.00 × 10^1^
	σ, Ω	2.74 × 10^0^	1.72 × 10^0^	3.74 × 10^0^
	V, %	45.6	25.3	37.4

* $\bar{x}$—average, σ—standard deviation, V—coefficient of variation.

**Table 8:** Results of average values of surface resistances in the manufactured electrodes.

Measure Variant	Statistical Measure	Initial Resistance	Resistance after Five Washes	Resistance after 900 Friction Strokes	Resistance after 500 Stretching Cycles
commercial electrode	$\bar{x}$, Ω	3.51 × 10^5^	-	-	-
	σ, Ω	6.17 × 10^5^	-	-	-
	V, %	175.9	-	-	-
cotton knitwear MWCNT printed	$\bar{x}$, Ω	9.10 × 10^1^	1.89 × 10^2^	1.23 × 10^2^	1.26 × 10^2^
	σ, Ω	9.01 × 10^0^	9.03 × 10^1^	4.02 × 10^1^	1.78 × 10^1^
	V, %	9.9	47.8	32.7	14.1
cotton knitwear MWCNT and GO printed	$\bar{x}$, Ω	2.66 × 10^1^	2.68 × 10^2^	6.93 × 10^1^	6.24 × 10^1^
	σ, Ω	5.89 × 10^0^	6.97 × 10^1^	1.98 × 10^1^	1.53 × 10^1^
	V, %	22.1	26.0	28.6	24.4
cotton knitwear X-Static embroidered	$\bar{x}$, Ω	1.82 × 10^−1^	1.97 × 10^−1^	1.36 × 10^−1^	2.02 × 10^−1^
	σ, Ω	8.35 × 10^−2^	5.19 × 10^−2^	1.38 × 10^−1^	1.37 × 10^−1^
	V, %	45.9	26.3	101.6	67.8
polyester fabric with MWCNT print	$\bar{x}$, Ω	5.60 × 10^1^	2.04 × 10^2^	1.29 × 10^2^	8.60 × 10^1^
	σ, Ω	1.30 × 10^1^	5.13 × 10^1^	6.68 × 10^0^	8.26 × 10^0^
	V, %	23.3	25.1	5.2	9.6
polyester knitwear with MWCNT and GO print	$\bar{x}$, Ω	6.26 × 10^1^	1.95 × 10^2^	1.04 × 10^2^	1.00 × 10^2^
	σ, Ω	5.99 × 10^0^	6.97 × 10^1^	1.98 × 10^1^	1.53 × 10^1^
	V, %	9.7	35.7	19.0	15.3
polyester knitwear X-Static embroidered	$\bar{x}$, Ω	8.00 × 10^−2^	9.00 × 10^−2^	2.42 × 10^−1^	1.92 × 10^−1^
	σ, Ω	4.60 × 10^−2^	4.34 × 10^−2^	2.69 × 10^−1^	1.82 × 10^−1^
	V, %	57.6	48.2	111.4	94.8
viscose knitwear with MWCNT print	$\bar{x}$, Ω	9.34 × 10^1^	1.66 × 10^2^	1.03 × 10^2^	1.08 × 10^2^
	σ, Ω	6.91 × 10^1^	3.39 × 10^1^	3.29 × 10^1^	1.56 × 10^1^
	V, %	74.0	20.4	31.9	14.4
viscose knitwear with MWCNT and GO print	$\bar{x}$, Ω	1.17 × 10^2^	1.85 × 10^2^	1.73 × 10^2^	1.48 × 10^2^
	σ, Ω	6.83 × 10^1^	5.59 × 10^1^	1.18 × 10^2^	1.73 × 10^1^
	V, %	58.2	30.2	68.2	11.7
viscose knitwear X-Static embroidered	$\bar{x}$, Ω	1.16 × 10^−1^	1.50 × 10^−1^	5.60 × 10^−2^	1.22 × 10^−1^
	σ, Ω	1.15 × 10^−1^	1.08 × 10^−1^	1.85 × 10^−2^	1.54 × 10^−1^
	V, %	99.1	72.0	33.0	126.2

* $\bar{x}$—average, σ—standard deviation, V—coefficient of variation.

## Data Availability

Not applicable.
